# Curcumin Exerted Neuroprotection against Ozone-Induced Oxidative Damage and Decreased NF-*κ*B Activation in Rat Hippocampus and Serum Levels of Inflammatory Cytokines

**DOI:** 10.1155/2018/9620684

**Published:** 2018-12-30

**Authors:** Sendar Daniel Nery-Flores, María Luisa Mendoza-Magaña, Mario Alberto Ramírez-Herrera, José de Jesús Ramírez-Vázquez, Marina María de Jesús Romero-Prado, César Ricardo Cortez-Álvarez, Abraham Alberto Ramírez-Mendoza

**Affiliations:** ^1^Laboratorio de Neurofisiología, Departamento de Fisiología, Centro Universitario de Ciencias de la Salud, Guadalajara, Jalisco, Mexico; ^2^Departamento de Farmacobiología, Centro Universitario de Ciencias Exactas e Ingenierías, Universidad de Guadalajara, Guadalajara, Jalisco, Mexico

## Abstract

Ozone is a harmful tropospheric pollutant, causing the formation of reactive oxygen and nitrogen species that lead to oxidative damage in living beings. NF-*κ*B can be activated in response to oxidative damage, inducing an inflammatory response. Nowadays, there are no reliable results that consolidate the use of antioxidants to protect from damage caused by ozone, particularly in highly polluted cities. Curcumin has a strong antioxidant activity and is a potent inhibitor of NF-*κ*B activation with no side effects. The aim of this study is to evaluate the effect of curcumin in preventive and therapeutic approaches against oxidative damage, NF-*κ*B activation, and the rise in serum levels of IL-1*β* and TNF-*α* induced by acute and chronic exposure to ozone in rat hippocampus. One hundred male Wistar rats were distributed into five groups; the intact control, curcumin-fed control, the ozone-exposed group, and the preventive and therapeutic groups. These last two groups were exposed to ozone and received food supplemented with curcumin. Lipid peroxidation was determined by spectrophotometry, and protein oxidation was evaluated by immunodetection of carbonylated proteins and densitometry analysis. Activation of NF-*κ*B was assessed by electrophoretic mobility shift assay (EMSA), and inflammatory cytokines (IL-1*β* and TNF-*α*) were determined by ELISA. Curcumin decreased NF-*κ*B activation and serum levels of inflammatory cytokines as well as protein and lipid oxidation, in both therapeutic and preventive approaches. Curcumin has proven to be a phytodrug against the damage caused by the environmental exposure to ozone.

## 1. Introduction

Air pollution is a direct consequence of industrialization and is defined as an abnormally high concentration of any hazardous gas or particulate matter [1]. Worldwide, millions of human beings are chronically exposed to highly polluted air far above the security limits established by the World Health Organization (WHO). Ozone (O_3_) is an allotrope of oxygen generated by photochemical reactions and has high oxidizing power. The tropospheric O_3_ is a product generated by intense sunlight radiation acting on nitrogen oxides and volatile organic compounds derived from combustion of fossil fuels; thus, it is produced in densely populated cities [2, 3].

Several studies have documented that the high oxidizing power of O_3_ affects human health causing pulmonary and cardiovascular dysfunction [4]. Furthermore, exposure to ozone is capable of affecting the central nervous system (CNS) especially in regions susceptible to oxidative stress such as the hippocampus [5]. The oxidizing activity of O_3_ in living beings occurs through sequential and overlapped processes where the nasal exposure and inhalation of O_3_ induce the formation of reactive oxygen and nitrogen species (RONS) that can cause damage in two main forms: (a) by affecting the olfactory mucosa where RONS diffuse through the olfactory tract and spread in the CNS and (b) by acting on the respiratory epithelium and causing alterations in cellular homeostasis and endothelial integrity, resulting in pulmonary dysfunction. RONS subsequently damage the blood-brain barrier (BBB) and reach the CNS, causing an oxidative state and neuroinflammation [6–8]. Additional damages include altered neurogenesis, reactive astrocytosis, reduced dendritic spines, neurotransmitter imbalance, increased activity of superoxide dismutase, DNA fragmentation, neuronal apoptosis, and cognitive, memory, and motor impairment [8–12]. Also, chronic exposure to O_3_ has been associated to an increased risk of certain neurodegenerative diseases, such as Alzheimer's and Parkinson's [13, 14].

Nuclear factor kappa B (NF-*κ*B) is a key transcriptional factor which is activated by reactive oxygen species (ROS) and inflammatory cytokines. Under normal conditions, NF-*κ*B is present in the cytoplasm coupled to its inhibitor (IkB*α*). On activation, IkB*α* undergoes phosphorylation by IKK and ubiquitination-dependent degradation by the proteosome, and then, NF-*κ*B (p65-p50 heterodimer) is released and translocates into the nucleus where it binds to DNA-response elements, resulting in an increased expression of proinflammatory and prooxidant genes, among others [15, 16]. Furthermore, NF-*κ*B promotes the expression of its own inhibitor, IkB*α*. After IkB*α* is synthesized, it is transported into the nucleus where it binds to the heterodimer blocking the binding to response elements [17].

Some studies suggest that the elimination of RONS should be accomplished with the administration of exogenous antioxidants without decreasing or even improving the activity of the endogenous antioxidant system [18, 19]. The use of natural antioxidants as complementary therapies has been proposed to limit the effects of RONS.

A natural alternative is the phytodrug named curcumin (CUR) which is isolated from the rhizome of *Curcuma longa* and chemically defined as diferuloylmethane. CUR has powerful antioxidant and anti-inflammatory activities, among others as well important [20, 21]. Furthermore, it has been documented that CUR modulates signal transduction and gene expression. Benefic effects of CUR are due to its interactions with growth factors, receptors, transcriptional factors, cytokines, enzymes, and genes that regulate apoptosis. It has been shown that CUR acts as a scavenger against RONS, and *in vivo*, CUR enhances the activity of superoxide dismutase, catalase, and glutathione peroxidase [20, 22, 23]. Such properties support its potential for preventive and therapeutic applications.

The aim of the present study was to analyze the neuroprotective effect exerted by CUR in preventive and therapeutic approaches against the increase of lipid peroxidation (LPO), protein oxidation (PO), activation of NF-*κ*B, in the rat hippocampus; and serum cytokines, IL-1*β* and TNF-*α*, induced by acute and chronic exposure to O_3_ as a model of oxidative stress.

## 2. Materials and Methods

### 2.1. Animals

Animals were treated in accordance with the guidelines and requirements of the World Medical Association, of the Declaration of Helsinki, and with the National Institutes of Health guide for the care and use of laboratory animals (NIH publication no. 8023, revised 1978) which are established in the Ethical Committee of the Health Science Center (CUCS, Universidad de Guadalajara). All analytical procedures used in the experiments were performed according to established guidelines. For this study, we used 100 male Wistar rats (*Rattus norvegicus*), 21 days old, weighing ≈130 g. We choose this age because it is well known that young and old animals are more susceptible than mature ones to lipid peroxidation [24]. Thus, young animals could be more important to protect than older ones. This could be based on the results reported by Calderón-Garcidueñas et al. [13, 25] which suggest that neurodegenerative markers are present in the brains of young human inhabiting highly polluted cities. Animals were kept under light/dark cycles 12 × 12 h, 22 ± 2°C, and relative humidity of 50–60% with free access to water and food (Prolab®RMH Laboratory Animal Diet, 2500 Rodent 5P14).

### 2.2. Diet

An alcoholic extract was prepared with commercial curcumin (Curcuma Kosher, Batch no. 09076). The concentration of CUR in the extract was determined by UV spectrophotometry at *λ* 230 nm, and the molecular identity was determined by the infrared spectrum compared with a CUR standard (Sigma Chemical Co., St. Louis, MO, USA). The food pellets were impregnated with the alcoholic extract; the ethanol was evaporated at 60°C for 4 hours, and the homogeneous distribution of CUR in the food pellets was corroborated by UV-spectrophotometry at *λ* 230 nm in the ethanolic extract obtained from samples of food pellets [26, 27]. The daily amount of CUR administered was approximately 5.6 mg/kg body weight in the food. This dose corresponds to a daily intake of 400 mg for humans in average.

### 2.3. Experimental Design

Animals were randomly distributed into ten experimental groups with ten rats each. All rats were subjected to an adaptation period of seven days before the beginning of the experiment. The adaptation was done to minimize the effect of human contact, food, and the lodging place in the experimental model. The design was established considering two periods of O_3_ exposure: an acute phase (A, 15 days) and a chronic one (C, 60 days). Also, the manner of exposure to O_3_ and the CUR supplementation in the experimental groups were defined as preventive (P) or therapeutic (T) for each period, considering their respective control groups. This design led to the following groups: the acute intact control (AIC) (*n* = 10) and the chronic intact control (CIC) (*n* = 10) groups that were exposed to O_3_-free air, without CUR; the CUR control groups that received the CUR supplementation, with no exposition to O_3_ in the same periods, are ACC (*n* = 10) and CCC (*n* = 10); and the O_3_ control groups that were exposed to 0.7 ppm of O_3_ during the same phases are AOC (*n* = 10) and COC (*n* = 10). The therapeutic groups were exposed to 0.7 ppm of O_3_ for 7 days and, subsequently, were fed with CUR until the end of the exposure time, covering the acute phase (AT) (*n* = 10) and chronic phase (CT) (*n* = 10). The preventive groups (AP, *n* = 10 and CP, *n* = 10) received supplemented food with CUR for 7 days prior to and during O_3_ exposure, in both phases.

### 2.4. Ozone Exposure

Animals were daily exposed to O_3_ for 4 h at a constant concentration of 0.7 ppm. The animals were exposed for 15 days for the acute phase and during 60 days for the chronic phase. Animals were placed in a hermetic acrylic chamber (65 × 25 × 45 cm L/H/D), which was connected to a gas premix chamber (40 × 24 × 45 cm). The premix chamber received O_3_ generated by a Certizon C100 apparatus (Sander Elektroapparatebau GmbH, Uetze, Germany), which was fed with medical-grade oxygen. The O_3_ generated was mixed with O_3_-free air to adjust an aforementioned concentration. The O_3_ concentration was monitored with a semiconductor sensor (ES-600, Ozone Solutions Inc., Hull, Iowa) to adjust the flow of oxygen and air needed for a proper atmosphere with a constant flow of 1.6–1.2 l/min. As part of the biosecurity actions, the O_3_ expelled from the chamber was inactivated with a neutralizing filter containing a solution of sodium nitrite, potassium carbonate, glycerol, methanol, and water before being released to the air.

### 2.5. Tissue Samples

After the exposure period was completed, animals were euthanized by an intraperitoneal injection of sodium pentobarbital at a dose of 36 mg/kg. Blood was extracted by intracardiac puncture from all rats, serum was separated, and an antiprotease cocktail was added to samples that were frozen at −80°C until use.

Five rats of each group, in the acute and chronic phases, (*n* = 5) were decapitated, one at a time, and the head was chilled on ice. Brains were dissected and two cuts were done in the stereotaxic coordinates of −6.04 to −2.80 bregma. Hippocampi were dissected from both hemispheres. The left hippocampi were 10% homogenized in PBS with 10 *μ*l/ml of butyl hydroxy toluene 0.5 M (BHT) for LPO assay. The right hippocampi were homogenized in PBS with antiproteases (EDTA, EGTA, leupeptin, aprotinin, bestatin, and PMSF) with 0.2 mM mercaptoethanol (Sigma Chemical Co., St. Louis, MO) for PO assay. Samples were stored at −80°C until processed. The protein concentration was determined using the micro-Bradford method (Cat. # 500-0201, Bio-Rad, Hercules, CA, USA), and absorbance was determined with a microplate reader (EZ Read 400, Biochrom, Miami, FL, USA) at *λ* 595 nm.

The remaining five rats of each group, in acute and chronic phases (*n* = 5), were anesthetized and decapitated. Both hippocampi were dissected and homogenized in 1.2 ml of lysis buffer (0.6% NP10, 0.15 M NaCl, 10 mM Tris pH 7.9, 1 mM EDTA, 0.1 mM EGTA, 0.5 mM 2-mercaptoethanol, and 0.5 mM PMSF). Immediately, the homogenate was incubated for 5 min on ice and centrifuged at 1250 g, at 4°C, for 5 min. Nuclear proteins, contained in the pellet, were resuspended in 100 *μ*l of cold extraction buffer (10 mM HEPES pH 7.9, 0.1 mM EGTA, 0.1 mM EDTA, 1.5 mM MgCl 2, 420 mM NaCl, 0.5 mM 2-mercaptoethanol, 0.5 mM PMSF, and 25% glycerol) and were incubated on ice for 20 min. Then, the nuclear membrane debris was removed by centrifugation at 1250 g for 5 min at 4°C. The supernatants containing the nuclear proteins were stored at −80°C until further analysis by electrophoretic mobility shift assay (EMSA).

### 2.6. Determination of Lipid Peroxidation

LPO was performed by determining the concentration of malondialdehyde (MDA) and 4-hydroxynonenal (4-HNE), according to the manufacturer's instructions (Cat. # FR12, Oxford Biomedical Res., Oxford, MI, USA). Samples were centrifuged at 3000 g for 5 min at 4°C, and 250 *μ*l of each sample was transferred to assay tubes. Then, 812.5 *μ*l of N-methyl-2-phenylindole was added, mixed, and incubated at 45°C for 40 min. Next, 187.5 *μ*l of methanesulfonic acid was added and samples were immediately chilled in an ice bath. Samples were incubated at 45°C for 45 min. Then, the reaction was stopped in an ice bath and tubes were centrifuged at 15000 g at 4°C for 15 min. Samples were kept on ice, and 200 *μ*l of supernatants was transferred in triplicate to a microplate of 96 wells, and absorbance was determined at *λ* 595 nm. The standard curve was prepared by adding 650 *μ*l chromogen solution to increasing concentrations of 1,1,3,3-tetrametoxipropane (0.315–10 nmol/ml).

### 2.7. Detection of Oxidized Protein

This assay was performed to detect the carbonyl groups in oxidized proteins, which reflect the oxidative damage caused by O_3_. We used the OxyBlot kit according to the manufacturer's instructions (Cat. # S7150, Merck Millipore Corp., Billerica, MA, USA). Briefly, samples were adjusted at 4 *μ*g of protein/*μ*l; then, 5 *μ*l of each sample was denaturalized with 5 *μ*l of 12% sodium dodecyl sulfate. Five samples of each group were derivatized with dinitrophenylhydrazine (DNPH) and a replica of samples reacted with the derivatization control solution. A stop solution was added, and proteins were separated by 10% PAGE in a mini-PROTEAN chamber (Bio-Rad, Hercules, CA, USA) at 100 V. Proteins were electrotransferred overnight to PVDF membranes at 25 V and 4°C. Membranes were blocked overnight with 5% nonfat milk in PBS and incubated with rabbit anti-DNPH (1 : 150). The reaction was detected with peroxidase-labeled anti-rabbit IgG, and oxidized protein bands were visualized with Immobilon Chemiluminescent HRP substrate (Millipore Corp., Billerica, MA, USA). Digital images were obtained and analyzed with the software Image Studio Lite Ver 5.2® to determine the value of integrated optical density (IOD) per sample and per group obtaining data for statistical analysis.

### 2.8. Electrophoretic Mobility Shift Assay for NF-*κ*B

NF-*κ*B activation was analyzed by EMSA. Five *μ*g of protein from the nuclear fraction was incubated with biotinylated double-stranded NF-*κ*B oligonucleotide 5′-TTGTTACAAGGGACTTTCCGCTGGGGACTTTCGGGAGGCGTGG-3′; underlining indicates the NF-*κ*B binding site, following supplier instructions (Cat. # 20148X, LightShift Chemiluminescent EMSA, Thermo Fisher Scientific). The DNA-protein complex was resolved on 6% nondenaturing polyacrylamide gel at 100 V in TBE (45 mM Tris-borate, 1 mM EDTA, pH 8.3). DNA-protein complexes were electrotransferred onto a nylon membrane (Hybond-XL Amersham Pharmacia Biotech), and DNA was crosslinked to the membrane with a transilluminator (UVP model 2UV) at 302 nm for 15 min. After crosslinking, the membranes were blocked for 15 min and then incubated with streptavidin-HRP conjugate for 15 min and reactive bands were detected by chemiluminescence. Blot images were digitally acquired with an HP ScanJet 4670 scanner, and densitometry analysis of images was performed with the GelQuant.Net software. The results were expressed as integrated optical density (IOD).

### 2.9. Enzyme-Linked Immunosorbent Assay (ELISA)

The concentration of IL-1*β* and TNF-*α* was determined by ELISA kits (Cat. # RLB00, Cat. # RTA00, R&D Systems, Minneapolis, MN, USA). Fifty micrograms of total protein from serum was used. Absorbance was measured with a microplate reader (EZ Read 400, Biochrom) at *λ* 492 nm, and the concentration of cytokines was determined and expressed in pg/ml.

### 2.10. Statistical Analysis

LPO data and cytokine levels were analyzed with one-way ANOVA and Tukey's test as post hoc. The data of oxidized proteins were analyzed by estimated marginal means (EMM) and with the Bonferroni post hoc test. The data obtained from NF-*κ*B activation was analyzed through nonparametric Kruskal-Wallis and Mann–Whitney *U* tests. Significant differences were considered for a value of *p* < 0.05. GraphPad Prism 6.01 software (GraphPad Software Inc., La Jolla, CA) was employed for all analyses.

## 3. Results

The results of the control groups AIC and CIC represent the basal oxidative damage without O_3_ exposure and without dietary supplementation. The results of the control groups ACC and CCC represent the oxidative damage without exposure to O_3_ and with dietary supplementation. These groups were used to establish differences with the control group exposed to O_3_ in the acute phase (AOC) and the chronic phase (COC) without dietary supplementation. Also, they were used to compare the neuroprotective effect of CUR observed in the preventive or the therapeutic modes.

### 3.1. Curcumin Decreased Lipoperoxidation Levels

**Figure 1:**
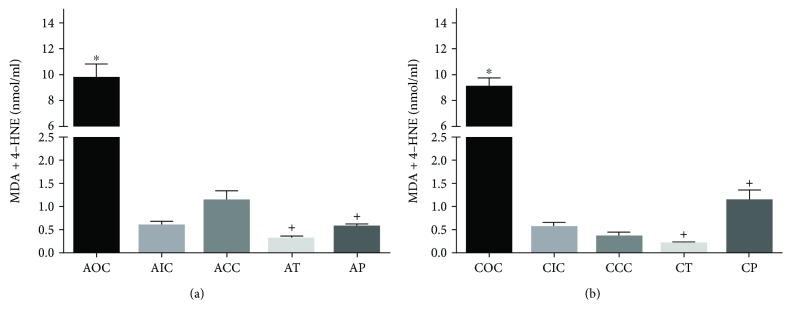
(a) Acute lipid peroxidation profile. AOC: acute O_3_ control; AIC: acute intact control; ACC: acute CUR control; AT: acute therapeutic; AP: acute preventive. ^∗^Statistical difference between the AOC vs AIC and ACC groups. ^+^Statistical difference between the AOC vs AT and AP groups. (b) Chronic lipid peroxidation profile. COC: chronic O_3_ control; CIC: chronic intact control; CCC: chronic CUR control; CT: chronic therapeutic; CP: chronic preventive. ^∗^Statistical difference between the COC vs CIC and CCC groups. ^+^Statistical difference between the COC vs CT and CP groups. Bars represent the concentration of MDA+4-HNE. Values are expressed as mean ± SEM.

The quantitation of LPO was performed with the spectrophotometric method in hippocampal homogenate samples as previously described. The one-way ANOVA test applied to LPO among all experimental groups was statistically significant (F[9, 40]=80.13; *p* < 0.001). As the maximum oxidative state in our design, the control group exposed to O_3_ showed a significantly increased level of LPO in the acute phase (AOC, 9.72 ± 2.36 nmol/ml; *p* < 0.001) in comparison with AIC (0.61 ± 0.18 nmol/ml) and ACC (1.13 ± 0.48 nmol/ml). The preventive and therapeutic supplementation of CUR in the acute phase caused a significant decrease of LPO (AT, 0.31 ± 0.11 nmol/ml; AP, 0.59 ± 0.12 nmol/ml; *p* < 0.001) in comparison with AOC (Figure 1(a)).

A similar pattern was observed in the chronic phase: the control group exposed to O_3_ showed a significantly increased concentration of MDA+4-HNE (COC, 9.03 ± 0.15 nmol/ml; *p* < 0.001), in comparison with the CIC (0.58 ± 0.21 nmol/ml) and CCC (0.36 ± 0.19 nmol/ml) groups. Furthermore, the diet supplemented with CUR caused a significant decrease in the concentration of MDA+4-HNE in the chronic phase (CT, 0.21 ± 0.06 nmol/ml and CP, 1.14 ± 0.47 nmol/ml; *p* < 0.001) in comparison with that of the COC group (Figure 1(b)).

### 3.2. Curcumin Reduced Protein Oxidation

**Figure 2:**
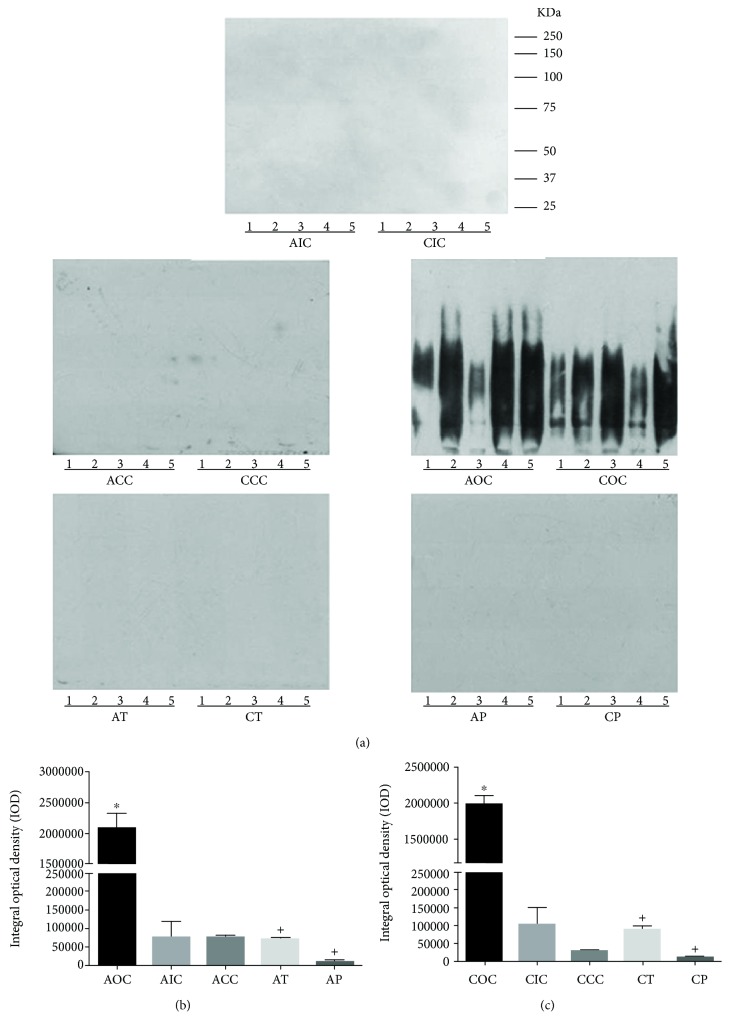
(a) Protein oxidation profile. Representative example of the control and experimental groups. Lanes are numbered for each sample used (*n* = 5). (b) Densitometry analysis of the protein oxidation profile in the acute phase. ^∗^Statistical difference between the AOC vs AIC and ACC groups. ^+^Statistical difference between the AOC vs AT and AP groups. (c) Densitometry analysis of the protein oxidation profile in the chronic phase. ^∗^Statistical difference between COC vs CIC and CCC groups. ^+^Statistical difference between the COC vs CT and CP groups. Values are expressed as mean ± SEM.

The protein carbonylation is an evidence of protein oxidation. The conjugation of DNPH with carbonyl residues was detected with antibodies against DNP. A representative example of the PO profile for each group is shown in Figure 2(a), which illustrates the immunodetection of carbonylated proteins in hippocampal homogenates. The statistical analysis of EMM showed that the IOD value of the AOC group had a significant increase in the PO profile (2078800 ± 435724; *p* < 0.001) in comparison with the AIC (75979 ± 75571) and ACC (77528 ± 6552) groups. When CUR was included as part of their diet in the therapeutic and preventive approaches, the IOD values were significantly reduced (71776 ± 6035 and 6437 ± 7412, respectively; *p* < 0.001) (Figure 2(b)).

In the chronic phase, the COC group showed a significant increase in the PO profile (2077276 ± 214471; *p* < 0.001) in comparison with the CIC and CCC groups (103843 ± 80734 and 30048 ± 3595, respectively). PO was decreased by the diet supplemented with CUR in the therapeutic group (CT, 89366 ± 15348) and in the preventive group (CP, 12644 ± 1686.19) when compared against the COC group (Figure 2(c)).

### 3.3. Curcumin Decreased the Activation and Translocation of NF-*κ*B

**Figure 3:**
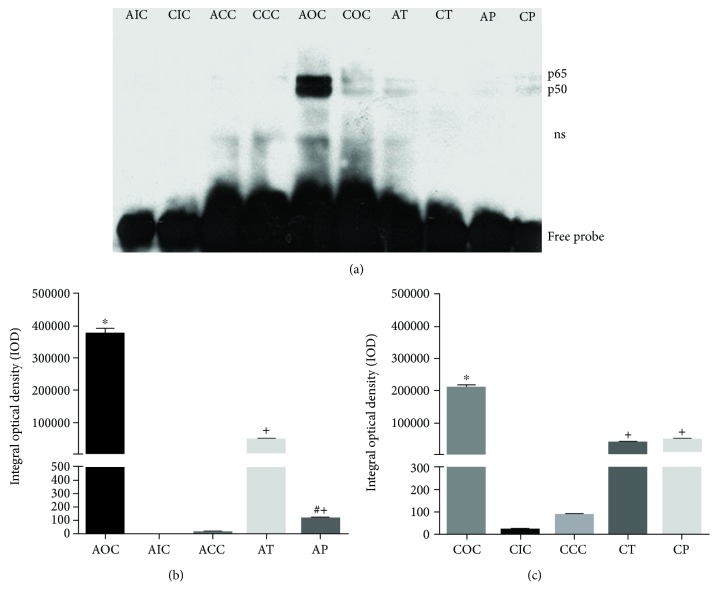
(a) Representative image of NF-*κ*B activation. AOC showed a strong binding to NF-*κ*B and remained activated at a lower level in the COC group. CUR showed an inhibitory effect against NF-*κ*B activation in the therapeutic and preventive approaches; ns: nonspecific binding. (b) Densitometry analysis of the NF-*κ*B activation profile in the acute exposure to O_3_. ^∗^Statistical difference between the AOC vs AIC and ACC groups. ^+^Statistical difference between AOC vs AT and AP groups. ^#^Statistical difference between the AT and AP groups. (c) Densitometry analysis of the NF-*κ*B activation profile in the chronic phase. ^∗^Statistical difference between the COC vs CIC and CCC groups. ^+^Statistical difference between the COC vs CT and CP groups. Values are expressed as mean ± SEM.

One of the major effects of O_3_ was the generation of RONS that caused the subsequent activation of NF-*κ*B. As we show in Figure 3(a), the acute response in rat hippocampus to this oxidant gas produced a strong activation of NF-*κ*B (AOC, 373727.63 ± 18362). The increase of NF-*κ*B activation was statistically significant compared with the those of AIC and ACC groups (0.00 ± 0.00 and 6.57 ± 2.63, respectively, *p* < 0.001). The ability of CUR to reverse or prevent the activation of NF-*κ*B in the acute phase was observed in the experimental groups AT (47333.40 ± 4081) and AP (109.13 ± 11.69). Furthermore, the AP group showed a significant decrease (*p* < 0.001) with respect to the AT group, suggesting that preventive administration of CUR has a greater effect on NF-*κ*B activation than the therapeutic administration after a 15-day exposure to O_3_ (Figure 3(b)). The NF-*κ*B activation in the chronic phase was increased in the COC group (207308.59 ± 11250) compared to the CIC and CCC groups (159.40 ± 17.70 and 777.82 ± 116, respectively, *p* < 0.001), demonstrating the ability of O_3_ to induce NF-*κ*B activation. Similarly, animals fed with CUR-supplemented diet in the therapeutic and preventive approaches had a significant decrease (*p* < 0.001) in NF-*κ*B activation, in both the CT and CP groups. Additionally, the CT and CP groups showed a similar activation of NF-*κ*B (Figure 3(c)). However, the COC showed a significant reduced NF-*κ*B activation compared to the AOC (*p* < 0.001).

### 3.4. Curcumin Reduced the Serum Concentration of IL-1*β* and TNF-*α*

**Figure 4:**
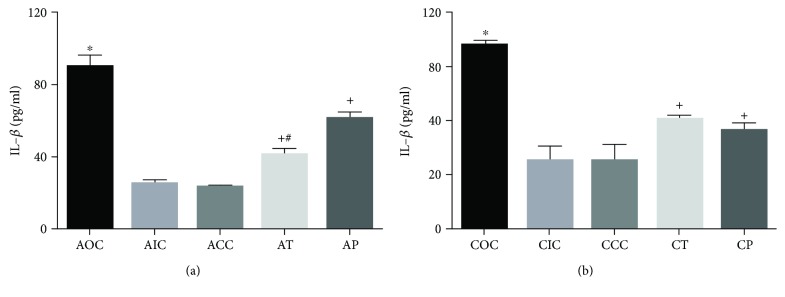
(a) Serum concentration of IL-1*β* in the acute exposure to O_3_. ^∗^Statistical difference between the AOC vs AIC and ACC groups. ^+^Statistical difference between the AOC vs AT and AP groups. ^#^Statistical difference between the AT and AP groups. (b) Serum concentration of IL-1*β* in the chronic exposure to O_3_. ^∗^Statistical difference between the COC and CIC and CCC groups. ^+^Statistical difference between the COC vs CT and CP groups. Values are expressed as mean ± SEM.

The activation of NF-*κ*B by acute and chronic exposure to O_3_ induced the rise of proinflammatory cytokine levels of IL-1*β* and TNF-*α*. The one-way ANOVA test applied to IL-1*β* and TNF-*α* among the experimental groups was statistically significant (F[9, 36]=33.51; *p* < 0.001 and F[9, 34]=6.24; *p* < 0.001, respectively). Furthermore, the diet supplementation with CUR exerted a strong reduction on cytokine serum levels. In the acute phase, the AOC group showed a significant increase in serum concentration for IL-1*β* (89.63 ± 6.78 pg/ml, *p* < 0.0001) compared with the groups AIC and ACC (24.88 ± 2.33 pg/ml and 23.83 ± 1.19 pg/ml, respectively). Meanwhile, the groups treated with CUR exhibited a significant decrease of IL-1*β* serum concentration (AT, 41.68 ± 3.41 pg/ml, *p* < 0.0001; AP, 61.49 ± 3.16 pg/ml, *p* < 0.0006) compared with the AOC group. Additionally, the AT group showed a significantly lower level than the AP group (*p* < 0.05, Figure 4(a)). In the chronic phase, the CIC and CCC groups did not show a significant difference between them (25.12 ± 5.15 pg/ml and 25.16 ± 5.96 pg/ml, respectively) nor among the levels found in the acute phase. On the contrary, the COC group displayed an increased concentration of IL-1*β* (67.87 ± 1.74 pg/ml, *p* < 0.0001) compared to the CIC and CCC groups. The effect of CUR in the chronic phase was significant as shown in data for the CT and CP groups (40.48 ± 1.52 pg/ml and 36.29 ± 2.92 pg/ml, respectively; *p* < 0.001) compared to the COC group (Figure 4(b)). When comparing the IL-1*β* level of the COC versus AOC groups, it seems to decrease in a time-dependent manner (*p* < 0.01), similar to that observed in the activation of NF-*κ*B.

**Figure 5:**
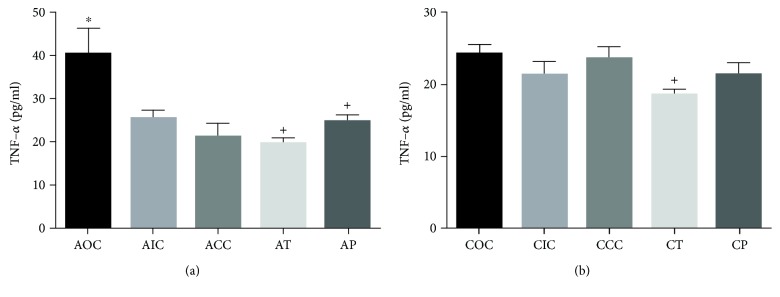
(a) Serum concentration of TNF-*α* in the acute exposure to O_3_. ^∗^Statistical difference between the AOC vs AIC and ACC groups. ^+^Statistical difference between the AOC vs AT and AP groups. (b) Serum concentration of TNF-*α* in the chronic exposure to O_3_. ^+^Statistical difference between the COC and CT groups. Values are expressed as mean ± SEM.

The effect of O_3_ was observed in the AOC group with a significant increase in the serum concentration of TNF-*α* (40.30 ± 6.14 pg/ml, *p* < 0.05) compared against the AIC and ACC groups (25.48 ± 1.95 pg/ml and 21.16 ± 3.24 pg/ml, respectively). The supplemented diet with CUR caused a decrease of TNF-*α* concentration in the AT and AP groups (19.67 ± 1.36 pg/ml; 24.73 ± 1.53 pg/ml, *p* < 0.05) compared with the AOC group (Figure 5(a)). The concentration of TNF-*α* in the chronic phase was not different among the AIC, ACC, COC, and CP groups, but the CT group showed a significantly lower level (18.55 ± 0.74 pg/ml, *p* < 0.05) when compared with the other groups (Figure 5(b)).

## 4. Discussion

In this work, the antioxidant and anti-inflammatory activities of CUR were evaluated in a model of oxidative stress caused by experimental exposure to O_3_. Here, we report that CUR exerted a neuroprotective effect in preventive or therapeutic approaches against oxidative damage, NF-*κ*B activation, and the rise of IL-1*β* and TNF-*α* serum levels caused by acute or chronic exposure to O_3_.

A considerable number of studies have evaluated and demonstrated the excellent antioxidant activity of CUR against damage induced by different oxidant substances [27–30] and as anti-inflammatory agent against the damage caused by the particulate matter [31, 32]. Oxidative stress is a common process that pollutants and other physical and chemical agents are able to induce [2]. Among air pollutants, O_3_ is by far the most powerful pollutant due its ubiquity, high reactivity, and oxidant power [33]. Oxidative stress is strongly related to chronic inflammation, and both processes are involved in the pathogenesis of chronic degenerative diseases and cancer [34]. Thus, it is important to reduce the impact of such harmful factors in human health. Some efforts have been made to reduce the impact of oxidative stress in experimental models using natural and synthetic antioxidants against the oxidative damage caused by O_3_ as taurine, tibolone, imipramine, and vitamin E. These molecules have demonstrated antioxidant activity, but their long-term administration could lead to the onset of undesirable side effects [35–38].

We propose the use of CUR, a natural diphenolic compound that has multiple desirable properties as a neuroprotective molecule based on its antioxidant and anti-inflammatory effects [21, 39]. CUR has been administered for long periods and at high doses (8 g/day) without adverse side effects [40, 41]. Previous studies have well demonstrated that CUR is able to cross the blood-brain barrier and is mainly concentrated in the hippocampus; therefore, CUR is able to carry out its activities in the CNS [42, 43].

It has been reported that acute and chronic O_3_ exposure causes oxidative stress and inflammation in the CNS, particularly in the hippocampus because it is a highly susceptible region to oxidative damage [5, 8]. The experimental model based on damage in the hippocampus induced by O_3_ acute and chronic exposure was corroborated by the oxidation of lipids and proteins.

Lipid peroxidation generates toxic aldehydes such as 4-HNE and MDA, which alter the structural and functional integrity of the plasma membrane that could trigger an inflammatory process in the CNS [44]. The concentration of these aldehydes is increased in several neurodegenerative diseases, which demonstrates its involvement in such pathological conditions [45]. Protein oxidation generated by O_3_ exposure occurs through a carbonylation process caused by ROS and reactive aldehydes, which induce covalent modification of proteins via nonenzymatic Michael addition. The carbonyl adducts lead to a dysfunctional behavior or loss of protein function that promotes the development of neuroinflammation and neurodegenerative diseases [46–48].

Our results show that the levels of lipid peroxidation and protein oxidation were increased in the rat hippocampus after acute exposure to O_3_ compared to controls. These oxidative levels were maintained in the chronic phase. However, other studies report that the oxidative damage caused by O_3_ increased progressively as a function of time [8, 49]. The difference observed in this effect may be attributable to the dose of O_3_ used in our study (0.7 ppm), compared to those used in other studies where the O_3_ dose was lower (0.25 ppm) and leading to an oxidative damage progressively increased in a time-dependent manner [8, 48]. Our data suggests that at the dose of 0.7 ppm, the endogenous antioxidant defenses had been overcome in the acute phase and this condition remained until the end of the chronic exposure.

We demonstrated that the dietary supplementation with CUR in the preventive and therapeutic approaches effectively decreased the oxidative damage to lipids and proteins in the CNS caused by acute or chronic exposure to O_3_. This effect may be due to direct or indirect antioxidant mechanisms. The direct mechanism occurs when CUR acts as a RONS scavenger molecule [50, 51]. The reaction of peroxyl radicals with CUR produces CUR-phenoxyl radicals yielding protective effect against lipoperoxidation generated by O_3_ exposure [52]. The indirect mechanism could be through the ability of CUR to induce the activation of Nrf2 to stimulate the expression of antioxidant enzymes that might play a protective role in the CNS against oxidative damage [53–55]. Additional studies are necessary to determine if the antioxidant effect of curcumin on oxidative damage by O_3_ is mediated in part by the activation of Nrf2.

NF-*κ*B plays a vital role in regulating the inflammation response in many diseases including brain injury and neurodegenerative diseases [56]. Exposure to O_3_ induces the formation of RONS and inflammatory cytokines in the lung tissue and the olfactory tract. In the CNS, these molecules are capable of activating NF-*κ*B that promotes the expression of proinflammatory genes [57, 58]. Our results showed an increase in the activation of NF-*κ*B in the acute phase of exposure while chronic exposure to O_3_ showed a decreased activation of NF-*κ*B in the rat hippocampus. This decreased activation of NF-*κ*B in our study is similar to that reported by Rivas et al. [48], where NF-*κ*B translocation occurred in the substantia nigra at 7 days of exposure and decreased after 60 days. The decrease of NF-*κ*B activation during chronic exposure could be due to a compensatory regulation that may involve the synthesis of I*κ*B*α*, the activation of Nrf2, the oxidative damage of NF-*κ*B p50 subunit, or the expression of anti-inflammatory cytokines [59–62].

CUR was able to significantly reduce the activation of NF-*κ*B in both the preventive and therapeutic approaches, in acute and chronic exposure. This demonstrates the ability of CUR to inhibit NF-*κ*B activation caused by O_3_. CUR acts on the signaling pathway of NF-*κ*B by inhibiting the activity of IKK and thus suppressing the phosphorylation and degradation of I*κ*B*α*; consequently, the nuclear translocation of NF-*κ*B is prevented [63, 64]. In addition, the scavenger activity of CUR could inhibit the activation of IKK by ROS [65]. In our experiments, we found that the greater effect occurred when CUR was administrated in the preventive mode in the acute phase. This could be related to the ability of CUR to activate the Nrf2 pathway previously to the oxidative insult, leading to the expression of antioxidant enzymes and, therefore, prevent the activation of NF-*κ*B by ROS [53, 66].

O_3_ inhalation activates alveolar macrophages through IL-1 receptor and Toll-like receptor 4, which in turn leads to the activation of NF-*κ*B; this induces an increased expression of inflammatory mediators such as IL-1, IL-6, and TNF-*α* [67, 68]. The high levels of IL-1*β* and TNF-*α* found in our experiments in rats exposed to O_3_ revealed a systemic inflammatory status that may predict an inflammatory process in the CNS [69–71]. As these cytokines are able to cross the BBB, they are capable of stimulating the activation of NF-*κ*B and increasing the neuroinflammation previously developed in situ [56]. The highest levels of IL-1*β* and TNF-*α* were observed in our study during the acute phase of O_3_ exposure. At the end of the chronic phase, the levels of IL-1*β* decreased, while TNF-*α* levels returned to the steady state; this phenomenon could be due to a compensatory anti-inflammatory response. A similar effect was reported by González-Guevara et al. [57] in a dynamically scalable O_3_ exposure model, where TNF-*α* decreased to basal levels in the cerebral cortex. This effect may be due to the regulation of the chronic inflammatory response. “Early-response cytokines,” such as IL-1*β* and TNF-*α*, increase during acute inflammation and begin to decrease due to the regulation exerted by anti-inflammatory cytokines such as IL-10 and IL-13, which interfere with the signaling pathway of NF-*κ*B and therefore reduce the production of these inflammatory mediators [62]. Additional studies are needed to determine whether the inflammatory regulation during the chronic exposure to O_3_ is due to the secretion of anti-inflammatory cytokines. In addition, the decrease in IL-1*β* and TNF-*α* levels in the chronic phase can be related to the lower activation of NF-*κ*B in the hippocampus due to a lower stimulation of the pathway by peripheral cytokines as shown in our results. The anti-inflammatory effect of CUR as a modulator for IL-1*β* and TNF-*α* has been reported elsewhere in a variety of experimental conditions [72–77]. In our model, the therapeutic and preventive administration of CUR reduced the concentration of IL-1*β* during acute and chronic exposure to O_3_. Moreover, the effect of CUR caused a significant decrease of TNF-*α* in the acute phase in the therapeutic and preventive administration modes. Thus, CUR exerts an anti-inflammatory activity by suppressing the transcription of proinflammatory cytokine genes through the NF-*κ*B signaling pathway [65, 78].

The results obtained in our experiments have led us to propose that the oxidative damage was established in the acute phase and remained unchanged throughout the chronic exposure phase; because the endogenous antioxidant system had been overcome at early time, such dynamics has been previously documented [5, 8, 48]. However, the inflammatory process has regulatory mechanisms that temporarily could lead to a diminution of the local or systemic inflammatory cytokines. Furthermore, this regulatory process could not be perpetuated and future insults could provoke a new imbalance manifested with an increase of inflammatory cytokine levels. To elucidate this point, we will design future experiments considering other strategies for the insult process.

Overall, it seems a very plausible idea that beneficial effects of CUR are more reliable in the preventive approach than in the therapeutic one [74, 76]. Therefore, it would be preferable to have a preventive protection against harmful factors especially if the protective strategy is free of side effects.

Our future work will explore whether early degenerative changes occur in the hippocampus of rats after a short-term exposure to O_3_ and whether CUR may prevent such deleterious changes.

## 5. Conclusions

The results presented in this study demonstrate the neuroprotective effect of CUR against the damage caused by exposure to O_3_. The administration of CUR decreased oxidative stress markers, such as LPO and PO, as well as the inflammatory profile by decreasing the activation of NF-*κ*B and inflammatory cytokines levels (IL-1*β* and TNF-*α*).

## Data Availability

The data related to lipid peroxidation, cytokine levels, densitometry determination, and images used to support the findings of this study are available from the corresponding author upon request.

## References

[1] Yang W., Omaye S. T. (2009). Air pollutants, oxidative stress and human health. *Mutation Research/Genetic Toxicology and Environmental Mutagenesis*.

[2] Lodovici M., Bigagli E. (2011). Oxidative stress and air pollution exposure. *Journal of Toxicology*.

[3] White W. H. (2009). Considerations in the use of ozone and PM(2.5) data for exposure assessment. *Air Quality, atmosphere & Health*.

[4] Raza A., Dahlquist M., Lind T., Ljungman P. L. S. (2018). Susceptibility to short-term ozone exposure and cardiovascular and respiratory mortality by previous hospitalizations. *Environmental Health*.

[5] Dorado-Martínez C., Paredes-carbajal C., Mascher D., Borgonio-Pérez G., Rivas-arancibia S. (2001). Effects of different ozone doses on memory, motor activity and lipid peroxidation levels, in rats. *The International Journal of Neuroscience*.

[6] Martínez-Lazcano J. C., González-Guevara E., Custodio V. (2018). Activity of nitric oxide synthase isoforms in acute brain oxidative damage induced by ozone exposure. *Nitric Oxide: Biology and Chemistry*.

[7] Pryor W. A., Houk K. N., Foote C. S. (2006). Free radical biology and medicine: it's a gas, man!. *American Journal of Physiology-Regulatory Integrative and Comparative Physiology*.

[8] Rivas-Arancibia S., Guevara-Guzmán R., López-Vidal Y. (2010). Oxidative stress caused by ozone exposure induces loss of brain repair in the hippocampus of adult rats. *Toxicological Sciences*.

[9] Avila-Costa M. R., Colín-Barenque L., Fortoul T. I. (1999). Memory deterioration in an oxidative stress model and its correlation with cytological changes on rat hippocampus CA1. *Neuroscience Letters*.

[10] Santiago-Lopez D., Bautista-Martinez J. A., Reyes-Hernandez C. I., Aguilar-Martinez M., Rivas-Arancibia S. (2010). Oxidative stress, progressive damage in the substantia nigra and plasma dopamine oxidation, in rats chronically exposed to ozone. *Toxicology Letters*.

[11] Rodríguez-Martínez E., Nava-Ruiz C., Escamilla-Chimal E., Borgonio-Perez G., Rivas-Arancibia S. (2016). The effect of chronic ozone exposure on the activation of endoplasmic reticulum stress and apoptosis in rat Hippocampus. *Frontiers in Aging Neuroscience*.

[12] Rivas-Arancibia S., Vazquez-Sandoval R., Gonzalez-Kladiano D., Schneider-Rivas S., Lechuga-Guerrero A. (1998). Effects of ozone exposure in rats on memory and levels of brain and pulmonary superoxide dismutase. *Environmental Research*.

[13] Calderón-Garcidueñas L., Reynoso-Robles R., Vargas-Martínez J. (2016). Prefrontal white matter pathology in air pollution exposed Mexico City young urbanites and their potential impact on neurovascular unit dysfunction and the development of Alzheimer's disease. *Environmental Research*.

[14] Calderón-Garcidueñas L., Mora-Tiscareño A., Ontiveros E. (2008). Air pollution, cognitive deficits and brain abnormalities: a pilot study with children and dogs. *Brain and Cognition*.

[15] Gupta S. C., Sundaram C., Reuter S., Aggarwal B. B. (2010). Inhibiting NF-*κ*B activation by small molecules as a therapeutic strategy. *Biochimica et Biophysica Acta (BBA) - Gene Regulatory Mechanisms*.

[16] Morgan M. J., Liu Z. G. (2011). Crosstalk of reactive oxygen species and NF-*κ*B signaling. *Cell Research*.

[17] Hoesel B., Schmid J. A. (2013). The complexity of NF-*κ*B signaling in inflammation and cancer. *Molecular Cancer*.

[18] Poljšak B., Fink R. (2014). The protective role of antioxidants in the defence against ROS/RNS-mediated environmental pollution. *Oxidative Medicine and Cellular Longevity*.

[19] Ndhlala A., Moyo M., Van Staden J. (2010). Natural antioxidants: fascinating or mythical biomolecules?. *Molecules*.

[20] Kunnumakkara A. B., Bordoloi D., Padmavathi G. (2017). Curcumin, the golden nutraceutical: multitargeting for multiple chronic diseases. *British Journal of Pharmacology*.

[21] Aggarwal B. B., Harikumar K. B. (2009). Potential therapeutic effects of curcumin, the anti-inflammatory agent, against neurodegenerative, cardiovascular, pulmonary, metabolic, autoimmune and neoplastic diseases. *The International Journal of Biochemistry & Cell Biology*.

[22] Gupta S. C., Kismali G., Aggarwal B. B. (2013). Curcumin, a component of turmeric: from farm to pharmacy. *BioFactors*.

[23] Barzegar A., Moosavi-Movahedi A. A. (2011). Intracellular ROS protection efficiency and free radical-scavenging activity of curcumin. *PLoS One*.

[24] Servais S., Boussouar A., Molnar A., Douki T., Pequignot J. M., Favier R. (2005). Age-related sensitivity to lung oxidative stress during ozone exposure. *Free Radical Research*.

[25] Calderón-Garcidueñas L., Leray E., Heydarpour P., Torres-Jardon R., Reis J. (2016). Air pollution, a rising environmental risk factor for cognition, neuroinflammation and neurodegeneration: the clinical impact on children and beyond. *Revue Neurologique*.

[26] Frautschy S. A., Hu W., Kim P. (2001). Phenolic anti-inflammatory antioxidant reversal of A*β*-induced cognitive deficits and neuropathology. *Neurobiology of Aging*.

[27] Canales-Aguirre A. A., Gomez-Pinedo U. A., Luquin S., Ramírez-Herrera M. A., Mendoza-Magaña M. L., Feria-Velasco A. (2012). Curcumin protects against the oxidative damage induced by the pesticide parathion in the hippocampus of the rat brain. *Nutritional Neuroscience*.

[28] Srivastava P., Yadav R. S., Chandravanshi L. P. (2014). Unraveling the mechanism of neuroprotection of curcumin in arsenic induced cholinergic dysfunctions in rats. *Toxicology and Applied Pharmacology*.

[29] Sharma C., Kaur J., Shishodia S., Aggarwal B. B., Ralhan R. (2006). Curcumin down regulates smokeless tobacco-induced NF-*κ*B activation and COX-2 expression in human oral premalignant and cancer cells. *Toxicology*.

[30] dos Santos Jaques J. A., Doleski P. H., Castilhos L. G. (2013). Free and nanoencapsulated curcumin prevents cigarette smoke-induced cognitive impairment and redox imbalance. *Neurobiology of Learning and Memory*.

[31] Zhang Z., Niu X., Lu C., Jiang M., Xiao G. G., Lu A. (2012). The effect of curcumin on human bronchial epithelial cells exposed to fine particulate matter: a predictive analysis. *Molecules*.

[32] Nemmar A., Subramaniyan D., Ali B. H. (2012). Protective effect of curcumin on pulmonary and cardiovascular effects induced by repeated exposure to diesel exhaust particles in mice. *PLoS One*.

[33] Genc S., Zadeoglulari Z., Fuss S. H., Genc K. (2012). The adverse effects of air pollution on the nervous system. *Journal of Toxicology*.

[34] Khansari N., Shakiba Y., Mahmoudi M. (2009). Chronic inflammation and oxidative stress as a major cause of age-related diseases and cancer. *Recent Patents on Inflammation & Allergy Drug Discovery*.

[35] Rivas-Arancibia S., Dorado-Martínez C., Borgonio-Pérez G. (2000). Effects of taurine on ozone-induced memory deficits and lipid peroxidation levels in brains of young, mature, and old rats. *Environmental Research*.

[36] Farfán-García E. D., Castillo-Hernández M. C., Pinto-Almazán R., Rivas-Arancibia S., Gallardo J. M., Guerra-Araiza C. (2014). Tibolone prevents oxidation and ameliorates cholinergic deficit induced by ozone exposure in the male rat hippocampus. *Neurochemical Research*.

[37] Mokoena M. L., Harvey B. H., Oliver D. W., Brink C. B. (2010). Ozone modulates the effects of imipramine on immobility in the forced swim test, and nonspecific parameters of hippocampal oxidative stress in the rat. *Metabolic Brain Disease*.

[38] Guerrero A. L., Dorado-Martinez C., Rodriguez A., Pedroza-Rios K., Borgonio-Perez G., Rivas-Arancibia S. (1999). Effects of vitamin E on ozone-induced memory deficits and lipid peroxidation in rats. *Neuroreport*.

[39] Cole G. M., Teter B., Frautschy S. A. (2007). Neuroprotective Effects of Curcumin. *Advances in Experimental Medicine and Biology*.

[40] Cooksey C. J. (2017). Turmeric: old spice, new spice. *Biotechnic & Histochemistry*.

[41] Gupta S. C., Patchva S., Aggarwal B. B. (2013). Therapeutic roles of curcumin: lessons learned from clinical trials. *The AAPS Journal*.

[42] Maiti K., Mukherjee K., Gantait A., Saha B. P., Mukherjee P. K. (2007). Curcumin–phospholipid complex: preparation, therapeutic evaluation and pharmacokinetic study in rats. *International Journal of Pharmaceutics*.

[43] Tsai Y.-M., Chien C.-F., Lin L.-C., Tsai T.-H. (2011). Curcumin and its nano-formulation: the kinetics of tissue distribution and blood–brain barrier penetration. *International Journal of Pharmaceutics*.

[44] Pizzimenti S., Ciamporcero E., Daga M. (2013). Interaction of aldehydes derived from lipid peroxidation and membrane proteins. *Frontiers in Physiology*.

[45] Perluigi M., Coccia R., Butterfield D. A. (2012). 4-Hydroxy-2-nonenal, a reactive product of lipid peroxidation, and neurodegenerative diseases: a toxic combination illuminated by redox proteomics studies. *Antioxidants & Redox Signaling*.

[46] Dkhar P., Sharma R. (2010). Effect of dimethylsulphoxide and curcumin on protein carbonyls and reactive oxygen species of cerebral hemispheres of mice as a function of age. *International Journal of Developmental Neuroscience*.

[47] Grimsrud P. A., Xie H., Griffin T. J., Bernlohr D. A. (2008). Oxidative stress and covalent modification of protein with bioactive aldehydes. *The Journal of Biological Chemistry*.

[48] Rivas-Arancibia S., Zimbrón L. F. H., Rodríguez-Martínez E., Maldonado P. D., Borgonio Pérez G., Sepúlveda-Parada M. (2015). Oxidative stress-dependent changes in immune responses and cell death in the substantia nigra after ozone exposure in rat. *Frontiers in Aging Neuroscience*.

[49] Pinto-Almazán R., Segura-Uribe J. J., Soriano-Ursúa M. A., Farfán-García E. D., Gallardo J. M., Guerra-Araiza C. (2018). Effect of tibolone pretreatment on kinases and phosphatases that regulate the expression and phosphorylation of Tau in the hippocampus of rats exposed to ozone. *Neural Regeneration Research*.

[50] Ak T., Gülçin İ. (2008). Antioxidant and radical scavenging properties of curcumin. *Chemico-Biological Interactions*.

[51] Trujillo J., Chirino Y. I., Molina-Jijón E., Andérica-Romero A. C., Tapia E., Pedraza-Chaverrí J. (2013). Renoprotective effect of the antioxidant curcumin: recent findings(). *Redox Biology*.

[52] Priyadarsini K. (2014). The chemistry of curcumin: from extraction to therapeutic agent. *Molecules*.

[53] González-Reyes S., Guzmán-Beltrán S., Medina-Campos O. N., Pedraza-Chaverri J. (2013). Curcumin pretreatment induces Nrf2 and an antioxidant response and prevents hemin-induced toxicity in primary cultures of cerebellar granule neurons of rats. *Oxidative Medicine and Cellular Longevity*.

[54] Liu Z., Dou W., Zheng Y. (2016). Curcumin upregulates Nrf2 nuclear translocation and protects rat hepatic stellate cells against oxidative stress. *Molecular Medicine Reports*.

[55] Dong W., Yang B., Wang L. (2018). Curcumin plays neuroprotective roles against traumatic brain injury partly via Nrf2 signaling. *Toxicology and Applied Pharmacology*.

[56] Shih R.-H., Wang C. Y., Yang C. M. (2015). NF-kappaB signaling pathways in neurological inflammation: a mini review. *Frontiers in Molecular Neuroscience*.

[57] González-Guevara E., Martínez-Lazcano J. C., Custodio V., Hernández-Cerón M., Rubio C., Paz C. (2014). Exposure to ozone induces a systemic inflammatory response: possible source of the neurological alterations induced by this gas. *Inhalation Toxicology*.

[58] Mumaw C. L., Levesque S., McGraw C. (2016). Microglial priming through the lung-brain axis: the role of air pollution-induced circulating factors. *The FASEB Journal*.

[59] Gan L., Johnson J. A. (2014). Oxidative damage and the Nrf2-ARE pathway in neurodegenerative diseases. *Biochimica et Biophysica Acta (BBA) - Molecular Basis of Disease*.

[60] Marshall H. E., Stamler J. S. (2001). Inhibition of NF-*κ*B by S-Nitrosylation. *Biochemistry*.

[61] Nishi T., Shimizu N., Hiramoto M. (2002). Spatial redox regulation of a critical cysteine residue of NF-*κ*B *in vivo*. *Journal of Biological Chemistry*.

[62] Ward P. A., Lentsch A. B. (1999). The acute inflammatory response and its regulation. *Archives of Surgery*.

[63] Bharti A. C., Donato N., Singh S., Aggarwal B. B. (2003). Curcumin (diferuloylmethane) down-regulates the constitutive activation of nuclear factor–*κ*B and I*κ*B*α* kinase in human multiple myeloma cells, leading to suppression of proliferation and induction of apoptosis. *Blood*.

[64] Jobin C., Bradham C. A., Russo M. P. (1999). Curcumin blocks cytokine-mediated NF-*κ*B activation and proinflammatory gene expression by inhibiting inhibitory factor I-*κ*B kinase activity. *Journal of Immunology*.

[65] Singh S., Aggarwal B. B. (1995). Activation of transcription factor NF-*κ*B is suppressed by curcumin (diferuloylmethane). *Journal of Biological Chemistry*.

[66] Scapagnini G., Colombrita C., Amadio M. (2006). Curcumin activates defensive genes and protects neurons against oxidative stress. *Antioxidants & Redox Signaling*.

[67] Park J.-W., Taube C., Swasey C. (2004). Interleukin-1 receptor antagonist attenuates airway hyperresponsiveness following exposure to ozone. *American Journal of Respiratory Cell and Molecular Biology*.

[68] Connor A. J., Laskin J. D., Laskin D. L. (2012). Ozone-induced lung injury and sterile inflammation. Role of Toll-like receptor 4. *Experimental and Molecular Pathology*.

[69] Rivest S. (2001). How circulating cytokines trigger the neural circuits that control the hypothalamic–pituitary–adrenal axis. *Psychoneuroendocrinology*.

[70] Johnston R. A., Mizgerd J. P., Flynt L., Quinton L. J., Williams E. S., Shore S. A. (2007). Type I interleukin-1 receptor is required for pulmonary responses to subacute ozone exposure in mice. *American Journal of Respiratory Cell and Molecular Biology*.

[71] Nguyen M. D., Julien J.-P., Rivest S. (2002). Innate immunity: the missing link in neuroprotection and neurodegeneration?. *Nature Reviews Neuroscience*.

[72] Ni H., Jin W., Zhu T. (2015). Curcumin modulates TLR4/NF-*κ*B inflammatory signaling pathway following traumatic spinal cord injury in rats. *The Journal of Spinal Cord Medicine*.

[73] Wang J., Wang H., Zhu R., Liu Q., Fei J., Wang S. (2015). Anti-inflammatory activity of curcumin-loaded solid lipid nanoparticles in IL-1*β* transgenic mice subjected to the lipopolysaccharide-induced sepsis. *Biomaterials*.

[74] Sorrenti V., Contarini G., Sut S. (2018). Curcumin prevents acute neuroinflammation and long-term memory impairment induced by systemic lipopolysaccharide in mice. *Frontiers in Pharmacology*.

[75] Guo Y.-Z., He P., Feng A.-M. (2017). Effect of curcumin on expressions of NF-*κ*Bp65, TNF-*α* and IL-8 in placental tissue of premature birth of infected mice. *Asian Pacific Journal of Tropical Medicine*.

[76] Kumar P., Sulakhiya K., Barua C. C., Mundhe N. (2017). TNF-*α*, IL-6 and IL-10 expressions, responsible for disparity in action of curcumin against cisplatin-induced nephrotoxicity in rats. *Molecular and Cellular Biochemistry*.

[77] Kelany M. E., Hakami T. M., Omar A. H. (2016). Curcumin improves the metabolic syndrome in high-fructose-diet-fed rats: role of TNF-*α*, NF-*κ*B, and oxidative stress. *Canadian Journal of Physiology and Pharmacology*.

[78] Yu Y., Shen Q., Lai Y. (2018). Anti-inflammatory effects of curcumin in microglial cells. *Frontiers in Pharmacology*.

